# Laparoscopic resection of retroperitoneal intra-psoas muscle schwannoma: A case report and extensive literature review

**DOI:** 10.1016/j.ijscr.2020.07.065

**Published:** 2020-07-29

**Authors:** Awadh AlQahtani, Mohammed N. AlAli, Safaa Allehiani, Sulaiman AlShammari, Hussein Al-Sakkaf, Maria A. Arafah

**Affiliations:** a Department of Surgery, College of Medicine, King Saud University, Medical City, King Khalid University Hospital, Riyadh, Saudi Arabia; b Department of Radiology, King Khalid University Hospital, King Saud University, Riyadh, Saudi Arabia; c Department of Pathology, King Saud University, Riyadh, Saudi Arabia

**Keywords:** Schwannoma, Neurilemmoma, Psoas muscle, Laparoscopic anterior approach, Review

## Abstract

**Introduction:**

Soft tissue tumors of Schwann cells in either the peripheral or cranial nerves are known as schwannomas or also as neurilemmomas. Limited data exists regarding psoas muscle schwannoma in the literature. Therefore, we have done an extensive literature review and found a total number of 46 cases of intra-psoas schwannoma including our case, which are summarized in this article along with reporting our new case.

**Presentation of case:**

We report a very rare case of primary left intra-psoas muscle schwannoma in a 39-year-old Saudi gentleman, smoker with an unremarkable medical and surgical history. The patient presented to the emergency department with exacerbated chronic lower abdominal pain since almost 2 years. After conforming the diagnosis, the patient was admitted electively and underwent uneventful laparoscopic hand-assisted resection.

**Discussion:**

Schwannomas are neurogenic tumors which known to be solitary, benign, well circumscribed, encapsulated, and slow-growing tumors. Multiple imaging modalities are used to detect such a tumor. Although there is no consensus on the best surgical approach, endoscopic mini-laparotomy, laparoscopy (anterior [as in our case] or lateral), and robotic resection were reported with good outcomes.

**Conclusion:**

Retroperitoneal schwannoma and intra-psoas muscle variant are a rare entity, with slow growing rate and risk of malignant transformation. Therefore, high index of suspicion, good follow-up, and large-cohort studies are encouraged.

## Highlights

• Retroperitoneal schwannoma is a rare disease and needs a high index of suspicion to be diagnosed.
• Generally, retroperitoneal schwannomas are known to be non-sensitive either to radiation or to chemotherapy.
• Retroperitoneal schwannomas therefore, requiring complete surgical excision with negative margins.
• In retroperitoneal schwannomas, due to the presence of multiple surgical approaches and different presentations, surgical access should be individualized.

## 1 Introduction

Soft tissue tumors of Schwann cells in either the peripheral or cranial nerves are known as schwannomas or also as neurilemmomas. They predominantly affect female patients from the third to the fifth decade of life with a highly nonspecific presentation [1,2]. They are rare tumors and retroperitoneal types are even rarer (only 0.3–3%, except in cases of von Recklinghausen’s disease), especially schwannoma of psoas muscle which is extremely rare, showing limited reported cases worldwide with rarely malignant transformation [3,5]. The cornerstone in the management of retroperitoneal schwannomas is complete surgical excision as it is nonsensitive to radiation and chemotherapy [2,3]. This project has been reported in line with the SCARE criteria [8].

In this research article, we present a summarized extensive literature review as well as reporting a very rare case of primary left intra-psoas muscle schwannoma treated by anterior laparoscopic hand-assisted resection.

## 2 Presentation of case

A 39-year-old Saudi gentleman, smoker with an unremarkable medical, surgical and Family history, presented to the emergency department with exacerbated chronic lower abdominal pain since almost 2 years. The pain was mainly in the left lower quadrant, intermittent, pressure-like, and aggravated by sudden movement or lifting objects, which was improved but not relieved by analgesia. No urinary symptoms, signs of lower GI bleeding, obstruction, malignancy, history of irritable bowel syndrome or inflammation were found.

**Fig. 1:**
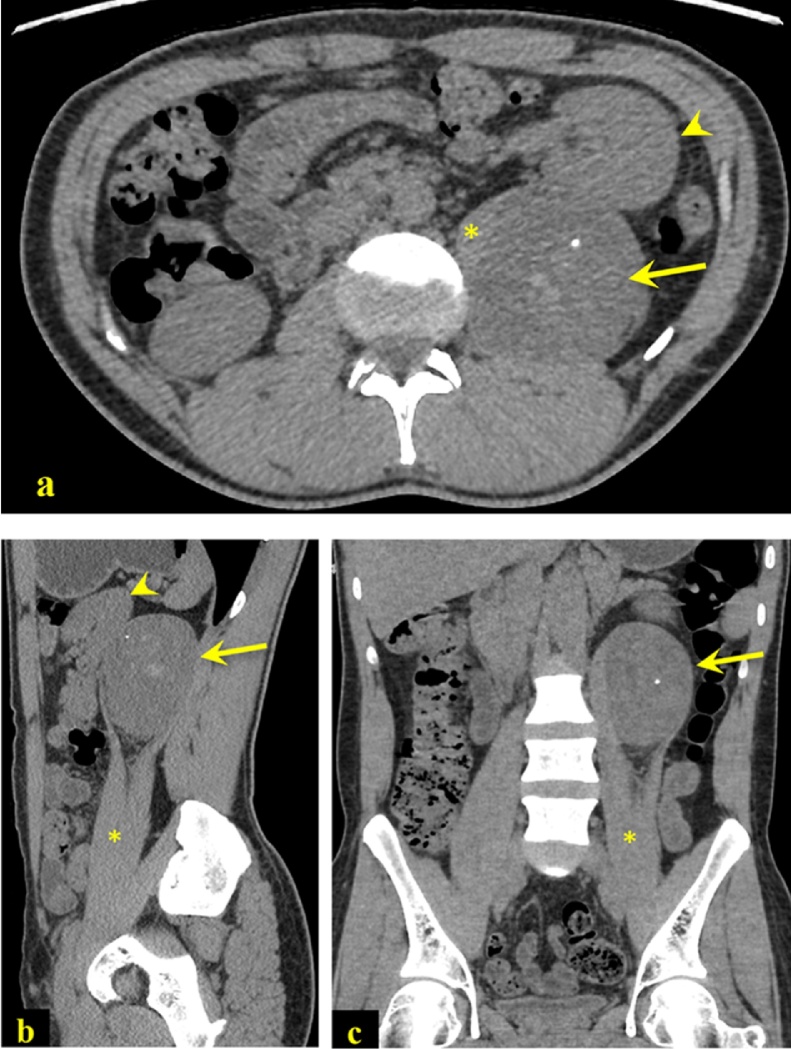
Axial (1a), sagittal (1b), and coronal (1c) CT scan of the abdomen without IV contrast obtained initially for renal colic. There is a well-defined oval paraspinal soft tissue mass (arrow) within the left psoas muscle (asterisk). The mass is heterogeneously hyperdense with foci of calcifications. The left kidney is displaced anteriorly (arrowhead).

**Fig. 2:**
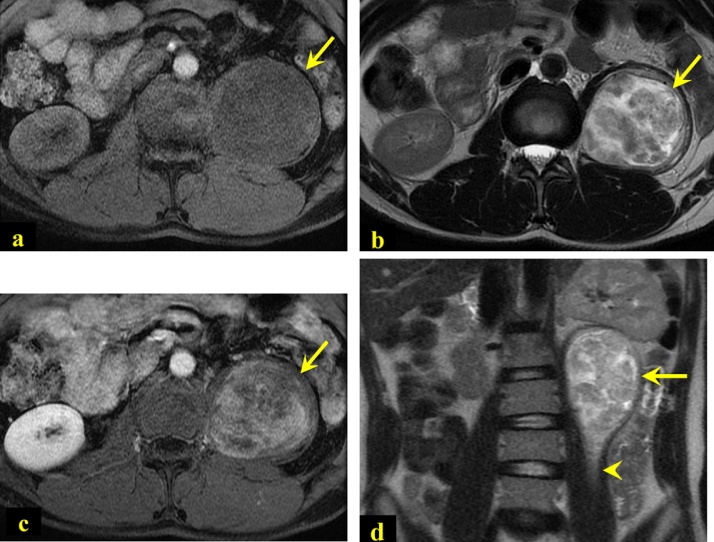
Axial T1 (2a), T2 (2b), and post-contrast T1-weighted (2c) MRI images showed well-defined T1 hypointense, T2 heterogeneous hyperintense, and heterogeneously enhancing mass in the posterior aspect of the left psoas muscle (arrow). Coronal T2-weighted image (2d) showed fat split sign (arrowhead) indicating intramuscular location of the mass.

**Fig. 3:**
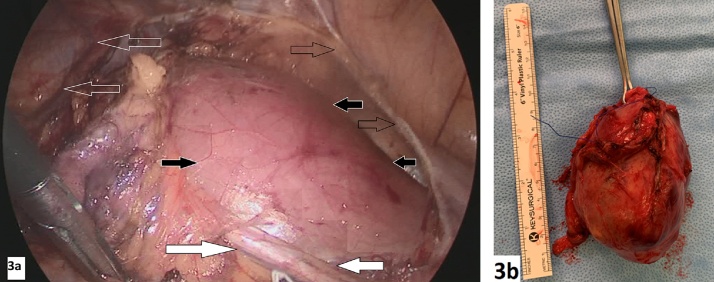
(3a) Laparoscopic view of the left side of the intra-abdominal cavity showing huge enlargement in left psoas muscle (solid black arrow), left ureter (solid white arrow), left descending colon mobilization sit (non-solid black arrow), and lower pole of left kidney (non-solid white arrow). (3b) The largest piece of the mass which measured 10 × 7.5 × 6.5 cm. The mass was oval with a smooth outer surface and a heterogenous nodular cut surface.

**Fig. 4:**
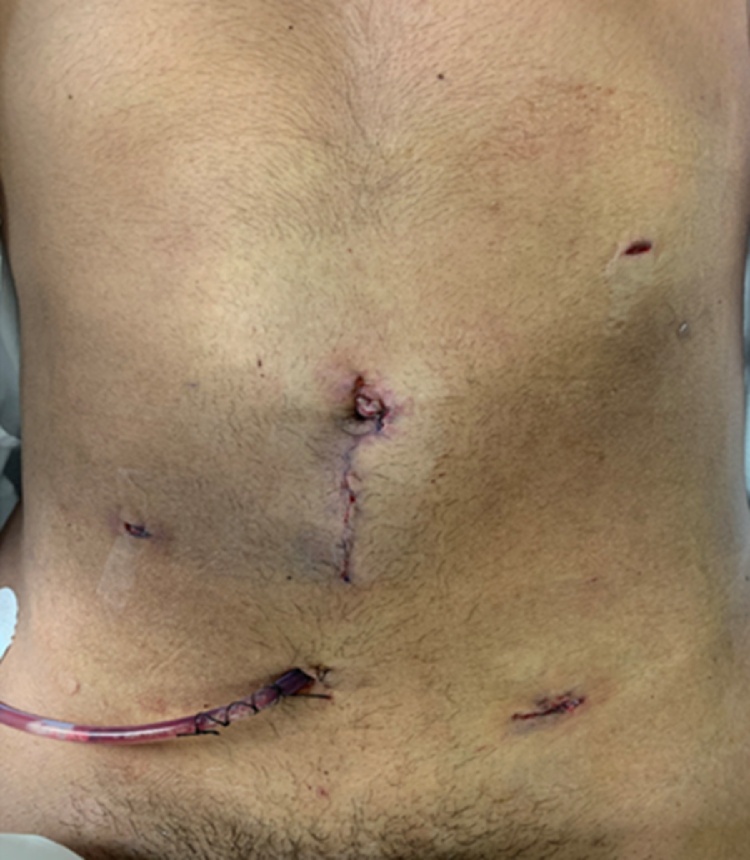
This picture demonstrated port sites and specimen extraction site. Four ports of size 5 mm were inserted as follows: supraumbilical area, left anterior axillar line, two fingers below the costal margin, and right anterior axillar line, near to superior anterior iliac spine and midline suprapubic area. 10 mm port was inserted in the left anterior axillar line, near to superior anterior iliac spine, in mid-lower abdominal laparotomy for specimen extraction.

On examination, the patient looked uncomfortable, in pain, but not in distress and vitally stable. The abdomen was nondistended, and there were significant tenders on deep palpation on the left lower quadrant without rebound tenderness. Routine lab and urine analyses were unremarkable. Unenhanced CT abdomen was done to rule out renal stones which showed left psoas muscle well-defined heterogeneous soft tissue mass (Fig. 1: a–c). He was referred to the surgical oncology unit and then underwent CT-guided left psoas mass biopsy which reported schwannoma with degenerative changes (ancient schwannoma). Magnetic resonance imaging (MRI) on the abdomen demonstrated a well-defined spherical-shape left intra-psoas muscle lesion measuring 6.4 × 8.5 × 6 cm on AP, CC, and TR, respectively (Fig. 2: a–d). After that, he was admitted electively and underwent a two-hour smooth anterior approach of laparoscopic hand-assisted resection of left psoas muscle mass using both splitting and cutting maneuvers through five ports performed by certified surgeon in surgical oncology and minimally invasive surgery (Fig. 3, Fig. 4).

The patient was placed in a semi-lateral decubitus position, the surgeon on the right side of the patient with his two assistants. The abdomen was accessed through the supraumbilical area by a 5 mm port. We proceed with an anterior intraperitoneal approach to the retroperitoneum through lateral colonic reflection. Initially, the patient underwent diagnostic laparoscopy then four more ports were placed after that we started with mobilization of the left colon and splenic flexure. Both left ureter and kidney (The anterior-inferior surface of the Gerota's fascia was exposed) were identified and preserved followed by visualization of the swollen left psoas muscle. Using Ligasure, blunt (longitudinal splitting), and cutting dissection were done to dissect the encapsulated mass from psoas muscle and spine was done till a plane was developed all around the mass. The tumor was gently eased out of its area and removed via an extension of the umbilical port. Surgicel was applied and hemostasis secured. After placement of closed suction drainage of the area, the left colon was repositioned on to the left side of the abdomen. The operative time was 167 min, and blood loss was about 43 mL.

**Fig. 5:**
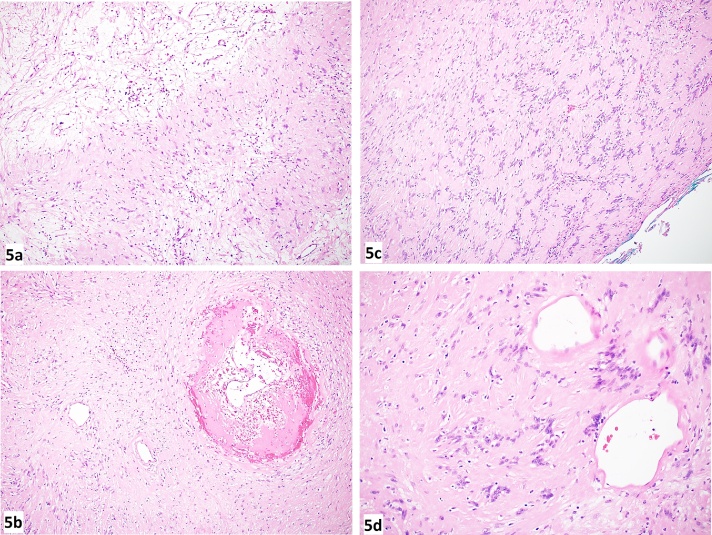
(5a) A photomicrograph showing the biphasic nature of the lesion (hypercellular Antoni A areas and hypocellular Antoni B areas) (H&E stain, x100 magnification). (5b) Areas of hyalinization and hemorrhage were present (H&E stain, x100 magnification). (5c) The cells showed elongated wavy nuclei with several Verocay bodies (H&E stain, x100 magnification). (5d) Mild nuclear atypia was present along with hyalinization of several blood vessels (H&E stain, x200 magnification).

The patient was discharged on day two postoperatively after showing good bowel motion, taking a regular diet with a significant improvement in pain. The postoperative histopathological examination showed the same diagnosis (Fig. 5: a–d). During regular follow-up (one week then four weeks and twelve weeks postoperatively), the patient was doing well with no limitation in movement or significant pain and not requiring analgesia.

## 3 Discussion

Schwannomas are neurogenic tumors which are known to be solitary, benign (about 5% of benign soft-tissue neoplasms), well-circumscribed, encapsulated, and slow-growing tumors. Only trunks of cranial nerves I and II are not vulnerable to schwannoma if compared to other parts of the body [1,6]. The malignant transformation is extremely rare except in the presence of type 2 neurofibromatosis which ranges from 5 to 18%. The clinical manifestation is significantly nonspecific, mainly depending on the size and location of the mass [1,2]. Till date, there is no real incidence or summary concerning what is reported in the literature about intra-psoas muscle schwannoma. Therefore, we did an extensive (English and non-English) literature review, using PubMed, Google Scholar, and RefSeek, and found around 45 cases of intra-psoas muscle schwannoma in addition to our current case (giving a total of 46 cases), as listed in Table 1.

**Table 1:** Literature review including cases of retroperitoneal intra-psoas muscle schwannomas.

Author, year	N. cases	Age	Sex	Symptoms and Duration	Location	Level	Size (cm)	Management	Dx. (histology or radiology)	F/U (M.)	Complications	Fx.	Notes
Ilyas M., et al., 2018 [4]	1	25	M	2wks/left flank pain and burning micturition	Left	NA	4 × 4.3	Surgical removal	Benign schwannoma	NA	None	NA	–
Rajkumar J. S., et al., 2015 [7]	1	30	M	Frontal right thigh pain	Right	L2	4 × 5	Anterior laparoscopic excision	Benign schwannoma	NA	None	NA	–
Syred, D. R., 1952 [11]	1	31	M	Pain between the scapulae and in the Ieft loin (max. at morning)	Left	NA	NA	NA	Benign schwannoma	7	Paralysis of quadriceps, sensory loss (anterior) thigh & knee	Negative	VRD (Negative)
Johenning P. W., et al., 1973 [12]	2	45	M	2yrs/low back pain radiating down the left thigh	Left	NA	5 × 4	Left paramedian transperitoneal approach	Benign schwannoma	24	None	Negative	VRD (Negative)
		53	F	Bitemporal headaches with vertigo, tinnitus, vomiting, and diarrhea	Right	NA	12 × 8 × 7.5	Retroperitoneal approach	Benign schwannoma	NA	NA	Negative	VRD (Negative)
Rufus Green, 1984 [13]	1	35	F	6M/right sided abdominal pain	Right	NA	6.5 × 4	Retroperitoneal approach	Benign schwannoma	12	None	Negative	–
Claes, H., et al., 1987 [14]	1	29	M	2yrs/lumbar pain radiating to the inguinal region (max. at night)	Left	L4-S1	3 × 5	Surgical exploration	Benign schwannoma (Antoni A & B)	12	NA	Negative	VRD (Negative)
Levine E., et al., 1987 [15]	1	23	F	Abdominal pain	Right	NA	8	NA	Benign Schwannoma	14	None	NA	Radiological review article
Frijns R. J. M., et al., 1989 [16]	1	42	M	Left sided ischialgia, weakness in the leg, weight loss, and fatigue	Left	NA	8 × 5 × 4	Transabdominal operation	**Malignant schwannoma (Rhabdomyoblastic differentiation)**	20	Recurrent, incomplete resection	NA	Reoperation, died after 20 M.
Downey, D. B., et al., 1989 [17]	1	39	M	15yrs/history of low back pain	Left	NA	NA	Surgical removal	Benign schwannoma	NA	NA	NA	–
Kuyumcuoglu, U., et al., 1990 [18]	1	35	F	1yr/right flank pain radiating to the inguinal region	Right	NA	9.5	Surgical removal	Benign schwannoma	None	None	NA	Passed away later, No F/U
Vesa Perhoniemi et al., 1992 [19]	1	76	M	Lower abdominal pain and mass	Right	NA	7	Follow-up	Benign schwannoma (Antoni A & B)	30	NA	NA	–
Kazutoshi Hida et al., 1993 [20]	1	56	M	2yrs/history of insidious weakness in the right leg	Right	L3-L4	4 × 3 × 3	Retroperitoneal approach	Benign schwannoma	NA	NA	NA	–
Yoshinori Nishi, et al., 1998 [21]	1	70	M	Severe low back pain, right femoral neuralgia, weakness, and urinary infection	Right	L3-L5	5	Anterior surgical removal	Benign schwannoma (Antoni A & B)	NA	Minimal weakness	NA	–
Rajagopal K. L., et al., 2002 [22]	1	38	M	1yr/history of fullness in the left side of the abdomen	Left	NA	NA (huge)	Retroperitoneal approach	Benign schwannoma	3	None	NA	–
Eiji Takahashi et al., 2003 [23]	1	60	M	Epigastric pain and sagging left lower limb	Left	NA	7 × 5 × 5	Surgical removal	Benign schwannoma	12	None	NA	–
Daneshmand S., et al., 2003 [24]	1	50	F	Numbness radiating down the lateral aspect of her left leg (years)	Left	L2-3	11	Left extrapleural, extraperitoneal thoracoabdominal incision over the 10th rib	Benign schwannoma (Antoni A & B)	38	None	NA	–
D'Silva, Karl J., et al., 2003 [25]	1	56	F	Persistent back pain and abdominal pain	Left	L4-S1	7.5 × 6 × 5	Combined surgical approach (multiple levels of laminectomies and fasciectomy)	Benign schwannoma (Antoni A & B)	NA	Walking aid	NA	VRD (Negative)
Jae Woong Jang, et al., 2004 [26]	1	59	M	1yr/back pain and pain radiating to both legs	Left	L4-L5	3 × 2	Retroperitoneal approach (then adjuvant radiotherapy)	**Malignant schwannoma**	10	None	Negative	VRD (Negative)
Pollo C., et al., 2004 [27]	1	41	M	Chronic back pain and unexplained weight loss	Right	L1-L2	14	Bilateral L1 and L2 laminectomy and anterior transperitoneal approach	Benign schwannoma (Antoni A & B)	NA	NA	NA	–
Takemoto, Jun, et al., 2004 [28]	1	44	F	Epigastric discomfort	Left	NA	15 × 8 × 8	Surgical removal	Benign schwannoma	NA	NA	NA	–
Liu Y. W., et al., 2007 [29]	1	35	M	Incidental	Right	NA	5	Surgical removal	Benign schwannoma	6	None	NA	–
Chun-Yu Fu, et al., 2008 [30]	1	57	M	Incidental	Right	L4-L5	3 × 3 × 2.6	Surgical removal	Benign schwannoma	6	None	NA	–
Muramatsu K., et al., 2008 [31]	2	77	F	Firm mass in the upper abdomin	Right	NA	6 × 7 × 6	Pararectal approach	Benign schwannoma	12	None	NA	–
		43	F	4yrs/lumbar pain and numbness of the left femur	Left	NA	5	Retroperitoneal approach	Benign schwannoma	NA	NA	NA	–
Morin S. H., et al., 2009 [32]	1	44	M	Incidental	Left	NA	NA	Conservative	Benign schwannoma	NA	NA	NA	Radiological review article
Sheng-sheng, X. U., 2009 [33]	2	NA	NA	NA	Right	NA	NA	NA	NA	NA	NA	NA	Radiological review article
Hsu Y. C., et al., 2010 [34]	1	43	F	Incidental	Right	L4	2.5	Surgical removal	Benign schwannoma	12	Decrease motor function	NA	–
Weil A. G., et al., 2011 [35]	1	77	F	Chronic right leg pain, paresthesias, and proximal right leg weakness	Right	L3-L4	NA	Minimally invasive approach (Spotlight tubular retractor)	Benign schwannoma	6	None	NA	–
Shimoda Y., et al., 2011 [36]	1	51	M	Chronic lower back pain and paresthesia in the lower left region	Left	L4-L5	6	Excision by Wiltse's approach	Benign schwannoma	12	None	NA	–
Seo I. Y., et al., 2011 [37]	1	46	M	Right abdominal discomfort (several months)	Right	NA	5.5 × 4.5	Laparoscopic resection	Benign schwannoma	NA	NA	NA	–
Kuriakose S., et al., 2014 [38]	1	19	F	2yrs/lower abdominal distention and right thigh pain	Right	NA	42 × 16 × 16	Exploratory laparotomy	Benign schwannoma (Antoni A & B)	24	Walking aid	NA	–
Lee, Seungcheol, et al., 2015 [39]	1	57	M	Chronic low back and right leg radicular pain	Right	L4	5.2 × 3.7 × 4.1	Direct lateral mini-open lateral retroperitoneal, trans-psoas approach	Benign schwannoma	12	None	NA	–
Formica, M., 2015 [40]	1	40	M	Slowly revealed pain, localized in the medial surface of the right knee, and hyposthenia of the right quadriceps muscle	Right	L3	NA	Radical surgical excision	**Malignant schwannoma**	NA	NA	NA	Radiological article
Sang Hoon Lee, et al., 2015 [41]	1	64	F	Incidental	Left	L3	4.8 × 4.8	Conservative	Benign schwannoma	NA	NA	NA	Radiological review article
Ramia J. M., et al., 2016 [42]	1	62	F	Severe post-hernioplasty pain in the entire inguinal region	Right	NA	2.6 × 2.5 × 3.5	Abdominal approach	Benign schwannoma (Antoni A & B)	6	None	NA	–
Vergara P., 2016 [43]	1	40	F	1yr/back pain, radiating to the left hip and groin, as well as mild weakness	Right	L1	NA	Dual approach (unilateral approach—midline, pars and facet sparing and no supplemental instrumentation)	Benign schwannoma	14	None	NA	–
Tej Kumar Y., et al., 2016 [44]	1	32	F	6M/recurrent (right) abdominal and lower limb pain (walking)	Right	NA	4 × 4	Surgical removal	Benign schwannoma (Antoni A & B)	NA	None	Negative	–
Zadro Z., et al., 2016 [45]	1	76	F	Left lower back pain radiating with parasthesia of lower limb (several months)	Left	L2-4	4.2 × 3.7 × 6	Posterior approach	Benign schwannoma	NA	None	NA	–
Benjamin, Carolina G., et al., 2016 [46]	1	38	M	2yrs/lumbar pain radiating distally with progressive motor weakness (feet)	Left	L4	1	Minimally invasive direct lateral trans-psoas approach	Benign schwannoma	None	None	NA	–
Halil Can Küçükyildiz, et al., 2017 [47]	1	43	F	2yrs/right leg pain	Right	L3	8 × 4 × 3	2 stages (L2 and L3 laminectomy and anterior retroperitoneal approach)	Benign schwannoma	NA	None	NA	–
Safaee M. M., et al., 2017 [48]	1	53	F	6yrs/progressively worsening low back and right anterior thigh pain	Right	L2-3	3.1 × 2.7 × 4.1	Lateral retroperitoneal trans-psoas approach	Benign schwannoma	NA	None	NA	–
Viswanath O., White, A. P., 2018 [49]	1	NA	NA	Right lower extremity pain and paresthesias, radiculopathy (femoral N. distribution)	Right	NA	12 × 6	NA	Benign schwannoma	NA	NA	NA	Anesthesia study
Zhai, H., et al., 2019 [50]	1	47	F	Incidental	Right	NA	NA	NA	Benign schwannoma	NA	NA	NA	Radiological review article
Current Study, 2020	1	39	M	Exacerbated chronic lower abdominal pain since almost 2 years	Left	NA	6.4 × 8.5 × 6	Anterior approach of laparoscopic hand-assisted resection	Benign schwannoma (Antoni A & B)	3	None	Negative	–
Total number of cases	46		Dx.: diagnosis, M: male, F: female, N: number, NA: not applicable, L: lumbar vertebrae, S: sacral vertebrae, Fx.: family histroy, F/U: follow-up, M.: months, VRD: von Recklinghausen's disease										

In 1952, Syred D. R. reported the first case of intra-psoas schwannoma, which was followed by a limited number of cases till today [11]. There was no difference between the male and female number of cases. The age ranged from 19 to 77 with no significant difference between decades. 27 cases indicated a right-side predominance. The majority of the patients presented with pain in the lower abdomen and lower back, numbness, weakness or paresthesia in the lower limb, or incidental finding of psoas mass. Some cases reported weight loss and were found to be malignant cases. There was significant variation in the size of tumors as the largest reported size was 42 × 16 × 16 cm while the smallest was 1 cm which did not correspond to the type of diagnosis. Multiple approaches where adopted, depending on the size, experience, and time (the most recent cases were more toward minimal invasive procedures). Most of the cases were benign (93.5%) except for 3 cases which were found to be malignant (6.5%), 2 on the left side and one on the right side, in patients aged 40 and above, with no reports of metastasis. Around half of the patients were followed up after the surgical intervention by a range of 3–38 months, with recurrence in one case only as it was malignant schwannoma and not completely resected. Some of the cases reported negative family history of schwannoma and von Recklinghausen's disease (VRD).

Most often, and like in our patient, incidental and delayed diagnosis of retroperitoneal mass is made which is most probably related to vague symptoms and large and flexible retroperitoneal space [2,3]. The differential diagnosis of retroperitoneal mass includes the following: benign (schwannomas, paragangliomas, angiomyolipoma), lymphoproliferative (Hodgkin’s), epithelial (kidney, adrenal, pancreas), metastatic (germ cell tumor, melanoma), pheochromocytoma, sarcoma (liposarcoma, leiomyosarcoma), and malignant fibrous histiocytoma [1,6].

A long fusiform structure located on both sides of the vertebral column and pelvis known as psoas muscle plays a significant role in human life. It consists of two origins: the deeper part comes from the first four lumbar vertebrae, while the superficial part comes from the lateral surface of the lower thoracic vertebrae and from adjacent intervertebral discs. The lumbar plexus is located in this area. Distally, it forms iliopsoas muscle by combining to iliacus muscle. During both movement and static states, the psoas major muscle is known to have a biomechanical and postural function. It is also involved in mood and stress disorders [9,10].

Multiple imaging modalities are used to detect such a tumor, like abdominal ultrasonography, CT abdomen, and MRI (the diagnostic modality of choice). If retroperitoneal tumors are suspected, MRI is highly recommended which offers a better delineation of the origin, extent, and internal composition of these lesions [3,6]. MRI is the gold standard image to establish the diagnosis of retroperitoneal schwannoma demonstrated as hypointense on T1 (Antoni type A tissue) and hyperintense on T2 (hypocellular Antoni B tissue) weighted MR images [2,3]. Schwannoma which is characterized by the presence of solid-cystic characteristics and degenerative histological changes, typical as in our case, is called ancient schwannoma. The accuracy of MRI in the recognition of tumor capsule is estimated to be 71% [2,5,7]. In the literature, it is not recommended to do CT-guided biopsy and fine needle aspiration to diagnose retroperitoneal schwannoma, but we did it in our case to confirm the diagnosis after MRI [2,3].

Based on the findings of pathology, histology, and immunohistochemistry, a definitive diagnosis is made. On histopathological examination, schwannoma has two types of Antoni areas, including Antoni A (interwoven bundles of bipolar cells in a well-organized, often palisading, pattern) and Antoni B (loosely textured pleomorphic cells), which can present separately or together. In our case, both are present. Immunohistochemistry stain for S-100 protein is a confirmatory test which is strongly expressed [[1], [2], [3],^6^].

Schwannoma is known to be nonsensitive both to radiation and chemotherapy. Therefore, complete surgical excision with negative margins (in both benign and malignant types) is the cornerstone in the management with good prognosis and low recurrence rate [3,5,6]. In the literature, there is no consensus on the best surgical approach, but endoscopic mini-laparotomy, laparoscopy (anterior [as in our case] or lateral), and robotic resection were reported with good outcomes. Malignant transformation and metastasis have been reported in the literature. Hence, good follow-up is highly recommended [2,3].

## 4 Conclusion

Up to date, the total number of reported intra-psoas schwannomas including our case is 46 cases. Generally, retroperitoneal schwannomas are rare, nonsensitive to chemotherapy or radiotherapy, and showing risk of malignant transformation. There is limited data about the incidence, signs and symptoms, and recurrence rate of intra-psoas muscle variant in the literature. Therefore, high index of suspicion, individualization of management, good follow-up, and large-cohort studies are required.

## Declaration of Competing Interest

All authors have nothing to disclose.

Awadh AlQahtani, Mohammed N Alali, Safaa Allehiani, Suliman AlShammari, Hussein Al-Sakkaf, and Maria A Arafah.

## Funding

All authors listed below have no source of funding to disclose.

## Ethical approval

There is no ethical approval was obtained as it’s a case report but a written consent was taken from the family as the patient passed away.

## Consent

Written informed consent was obtained from the patient for publication of this case report and accompanying images. A copy of the written consent is available for review by the Editor-in-Chief of this journal on request.

## Author contribution

Awadh AlQahtani: study concept, design, writing the paper.

Mohammed alali: data collection, data analysis, interpretation, writing the paper.

Safaa Allehiani: data collection, writing the paper.

Suliman Alshammari: data collection, writing the paper.

Hussein Al-Sakkaf: data interpretation (radiology part).

Maria A Arafah: data analysis interpretation (pathology part).

## Registration of research studies

Our paper is a case report, no registration was done for it.

## Guarantor

Awadh AlQahtani: dr_aq1@yahoo.com.

Mohammed alali: drmo7ammed2@gmail.com.

## Provenance and peer review

Not commissioned, externally peer-reviewed.
